# Oral bioavailability of the ether lipid plasmalogen precursor, PPI-1011, in the rabbit: a new therapeutic strategy for Alzheimer's disease

**DOI:** 10.1186/1476-511X-10-227

**Published:** 2011-12-05

**Authors:** Paul L Wood, Tara Smith, Nina Lane, M Amin Khan, Greg Ehrmantraut, Dayan B Goodenowe

**Affiliations:** 1 Dept. of Pharmacology, DeBusk College of Osteopathic Medicine, Lincoln Memorial University, 6965 Cumberland Gap Pkwy., Harrogate, TN 37752 USA; 2 R&D Dept., Phenomenome Discoveries Inc, 204-407 Downey Road, Saskatoon, SK, S7N 4L8 Canada

## Abstract

**Introduction:**

Docosahexaenoic acid (DHA) and DHA-containing ethanolamine plasmalogens (PlsEtn) are decreased in the brain, liver and the circulation in Alzheimer's disease. Decreased supply of plasmalogen precursors to the brain by the liver, as a result of peroxisomal deficits is a process that probably starts early in the AD disease process. To overcome this metabolic compromise, we have designed an orally bioavailable DHA-containing ether lipid precursor of plasmalogens. PPI-1011 is an alkyl-diacyl plasmalogen precursor with palmitic acid at sn-1, DHA at sn-2 and lipoic acid at sn-3. This study outlines the oral pharmacokinetics of this precursor and its conversion to PlsEtn and phosphatidylethanolamines (PtdEtn).

**Methods:**

Rabbits were dosed orally with PPI-1011 in hard gelatin capsules for time-course and dose response studies. Incorporation into PlsEtn and PtdEtn was monitored by LC-MS/MS. Metabolism of released lipoic acid was monitored by GC-MS. To monitor the metabolic fate of different components of PPI-1011, we labeled the sn-1 palmitic acid, sn-2 DHA and glycerol backbone with^13^C and monitored their metabolic fates by LC-MS/MS.

**Results:**

PPI-1011 was not detected in plasma suggesting rapid release of sn-3 lipoic acid via gut lipases. This conclusion was supported by peak levels of lipoic acid metabolites in the plasma 3 hours after dosing. While PPI-1011 did not gain access to the plasma, it increased circulating levels of DHA-containing PlsEtn and PtdEtn. Labeling experiments demonstrated that the PtdEtn increases resulted from increased availability of DHA released via remodeling at sn-2 of phospholipids derived from PPI-1011. This release of DHA peaked at 6 hrs while increases in phospholipids peaked at 12 hr. Increases in circulating PlsEtn were more complex. Labeling experiments demonstrated that increases in the target PlsEtn, 16:0/22:6, consisted of 2 pools. In one pool, the intact precursor received a sn-3 phosphoethanolamine group and desaturation at sn-1 to generate the target plasmalogen. The second pool, like the PtdEtn, resulted from increased availability of DHA released during remodeling of sn-2. In the case of sn-1 18:0 and 18:1 plasmalogens with [^13^C_3_]DHA at sn-2, labeling was the result of increased availability of [^13^C_3_]DHA from lipid remodeling. Isotope and repeated dosing (2 weeks) experiments also demonstrated that plasmalogens and/or plasmalogen precursors derived from PPI-1011 are able to cross both the blood-retinal and blood-brain barriers.

**Conclusions:**

Our data demonstrate that PPI-1011, an ether lipid precursor of plasmalogens is orally bioavailable in the rabbit, augmenting the circulating levels of unesterified DHA and DHA-containing PlsEtn and PtdEtn. Other ethanolamine plasmalogens were generated from the precursor via lipid remodeling (de-acylation/re-acylation reactions at sn-2) and phosphatidylethanolamines were generated via de-alkylation/re-acylation reactions at sn-1. Repeated oral dosing for 2 weeks with PPI-1011 resulted in dose-dependent increases in circulating DHA and DHA-containing plasmalogens. These products and/or precursors were also able to cross the blood-retinal and blood-brain barriers.

## Introduction

Alzheimer's disease (AD) is a devastating neurological disorder characterized by severe cognitive dysfunction. Diagnosis of AD is not simple and ultimately requires demonstration of AD pathology, namely the presence of argyrophilic plaques (amyloid deposition) and neurofibrillary degeneration of neurons in the cortex and hippocampus. However, AD pathology is often found in the brains of older persons with no cognitive impairment (NCI) [1-3]. These observations raise the question: "What is the best neuropathological correlate with cognitive decline in AD?" At this time, neuronal/brain atrophy [4] and cholinergic dysfunction [5,6] remain strongly correlated with cognitive decline in AD.

Brain shrinkage in AD is dramatic and is the result of decrements in myelin volume [7-10] and neuronal shrinkage that precedes neuronal losses late in the disease process. The importance of neuronal shrinkage has been demonstrated for the N. basalis.-cortex cholinergic projection [11-14] as well as for neurons in the amygdala [15] and the neocortex [16,17]. Of interest in this regard is the recent report that the brains from individuals with NCI, but a heavy AD pathological burden, suffer from less brain shrinkage than AD patients [18].

Decreases in critical brain plasmalogen levels for white and gray matter [19-22] are potentially the basis of these decreases in brain volume in AD. Since plasmalogens are essential structural phospholipids of myelin and neurons, decreases in these large phospholipid pools are probably responsible for hypo-myelination and the shrinkage of neurons that precedes cell loss. The liver, which is critical for the supply of plasmalogens and/or plasmalogen precursors to the brain [23], has decreased ability to synthesize these precursors in AD presumably, as a result of peroxisomal dysfunction [24] which has also been demonstrated in AD brain [25]. This peroxisomal dysfunction is further reflected in decreased circulating levels of plasmalogens in AD [26-28]. *In toto*, these data suggest that long-term alterations in plasmalogen synthesis/degradation result in decreased brain plasmalogen levels, a hallmark feature of AD.

To correct this metabolic deficiency, we designed a DHA-containing ether lipid precursor of plasmalogens that bypasses peroxisomes and has the ability to resupply critical plasmalogen precursors to the brain in AD. This ether lipid precursor, PPI-1011, can replace plasmalogens in cell lines and human lymphocytes with peroxisomal deficits [28,29] and can augment deficient circulating and tissue plasmalogens in the mouse cuprizone model of demyelination [28]. This report presents the pharmacokinetic data for this drug after oral dosing in rabbits.

## Materials and methods

### Drugs

PPI-1011 is an alkyl-diacyl plasmalogen precursor with palmitic acid at sn-1, DHA at sn-2 and lipoic acid at sn-3. The lipoic acid at sn-3 is required to stabilize the precursor and is rapidly cleaved by gut lipases prior to addition of the phosphoethanolamine at sn-3 [EC 2.7.8.1] in the gut epithelium and the liver. The final step in the generation of the 16:0/22:6 plasmalogen is desaturation of the sn-1 ether linkage [EC 1.14.99.19] in the endoplasmic reticulum of the gut epithelium and liver. Remodeling at sn-2 to generate other PtdEtn and PlsEtn occurs via the sequential actions of lipases removing the sn-2 fatty acid and acyltransferases replacing an alternate fatty acid. PPI-1038 is PPI-1011 labeled with [^13^C_16_]palmitic acid, [^13^C_3_]DHA, and [^13^C_3_]glycerol.

### PPI-1011 Time Course Study

All rabbit studies were conducted with the University of Saskatchewan Animal Care Committee approved protocol 20080106. Female New Zealand rabbits (1.8-2.5 kg; Charles River Labs.) were acclimated for 1 week prior to experimentation. PPI-1011 (200 mg/kg) was dispensed neat in hard gelatin capsules (size 2; Capsuline Inc., Pompano Beach, Fl.) using a pilling gun [30]. For the time course studies, rabbits were dosed with 200 mg/kg of PPI-1011 or corn oil and animals sacrificed with Euthanyl (pentobarbital sodium, 240 mg/ml; 1 ml/2.25 kg iv) at 0, 1, 3, 6, 12, 18, 24 and 48 hr. Blood was collected into tubes containing EDTA by cardiac puncture and centrifuged at 3500 rcf at 4°C for 10 mins. Plasma was removed and stored at -70°C for later analysis. Tissues were collected and flash frozen in liquid nitrogen and then stored at -70°C. Tissue harvests were conducted as 2 experiments with overlapping groups at 12 hrs (Exp. 1: 1, 3, 6, and 12 hrs; Exp. 2: 12, 18, 24 and 48 hrs). Controls were harvested at each timepoint.

### PPI-1038 Time Course Study

A female New Zealand rabbit was acclimated for 1 week prior to experimentation. PPI-1038 (100 mg/kg orally) was dispensed neat in a hard gelatin capsule. Serial blood samples were collected from the ear vein at times 0, 6, 24 and 48 hr. At 48 hr the rabbit was sacrificed with Euthanyl and tissues collected into TissueTubes (Covaris) and flash frozen in liquid nitrogen prior to storage at -70°C.

### PPI-1011 Dose Response Study

Six female New Zealand rabbits per group were acclimated for 1 week prior to experimentation. PPI-1038 (0, 10, 75, 200, 500 or 1000 mg/kg) was dispensed neat in hard gelatin capsules. Six hours after dosing, rabbits received Euthanyl and terminal blood collection was into tubes containing EDTA by cardiac puncture and plasma stored at -70°C for later analysis.

### PPI-1011 Repeated Dosing Study

Six female New Zealand rabbits per group were acclimated for 1 week prior to experimentation. PPI-1011 (0, 10, or 50 mg/kg) was dispensed neat in hard gelatin capsules once daily for 2 weeks. Twenty-four hours after the last dose rabbits received Euthanyl and terminal blood collection into tubes containing EDTA by cardiac puncture and tissue collection were conducted. Tissues were flash frozen in liquid nitrogen and both tissues and plasma were stored at -70°C for later analysis.

### Tissue Pulverization

Tissue samples in TissueTubes, plastic film-based pouches with screwtop plugs, were removed from dry ice and thoroughly frozen in liquid nitrogen before being placed into the Cryoprep CP02 (Covaris). The Cryoprep then freeze-fracture pulverized the tissue in < 1s using hammer and anvil technology. After being pulverized into a homogenous powder the sample was placed back in liquid nitrogen. A transfer tube was directly connected to the TissueTube which upon inversion allowed for easy transfer of the sample into extraction tubes without thawing.

### Plasmalogen Analyses

For plasmalogen analyses, 10 mg of tissue powder in 5 mL screw cap tubes were polytroned in 1 mL of PBS + 0.5 mL MeOH. In the case of plasma, 250 μL were vortexed with 1 mL of PBS + 0.5 mL MeOH. Next, 2 mL tert-butylmethylether were added to the plasma samples and tissue homogenates and the samples capped and shaken (1400 rpm) for 15 min at room temperature. The samples were then centrifuged for 8 min in a clinical centrifuge and 1 ml of the upper organic layer isolated for negative ion electrospray LC-MS/MS analyses of DHA, ethanolamine plasmalogens and phosphatidylethanolamines as reported previously [26-29]. For the labeled drug studies, the MRM transitions were adjusted to monitor the flux of the labeled palmitic acid, DHA and glycerol backbone. Background values were determined with time zero samples and subtracted from subsequent timepoints.

### Bismethythiohexanoic Acid (BMHA) Analyses

BMHA, the major metabolite of lipoic acid [31] was quantitated in rabbit plasma. The EDTA plasma (250 μL) was mixed with 1.2 ml of acetonitrile:MeOH:formic acid (800:200:2.4) containing [^2^H_3_]leucine (CDN Isotopes) as internal standard and centrifuged at 4°C and 25,000 × g for 30 min. Next, 400 μL of the supernatant were dried in a centrifugal evaporator. Alkylation of free carboxyl and amino groups was performed via heating at 80°C for 1 hr in 50 uL of pentafluorobenzyl bromide (5% in dimethylformamide) + 10 uL of diisopropylamine. The samples were next vortexed with 200 uL of hexane/ethyl acetate (3:2) followed by centrifugation at 25,000 × g and 24°C for 5 min. The clear supernatant containing the PFBB derivatives were analyzed by GC-MS with the [M-180]^- ^anions of 207.1 (BMHA) and 313.2 ([^2^H_3_]leucine) monitored under ammonia NCI. The GC-MS system was an Agilent 7890A GC and 5975C mass analyzer; the GC column was a 30 m HP-5MS with 0.25 mm ID and 0.25 μm film.

### Data Analyses

Data are presented as mean ± SEM. DHA, PtdEtns & PlsEtn were normalized to the housekeeping metabolite PtdEtn 16:0/18:0 while BMHA was expressed as nmoles/ml plasma. GC-MS analyses were performed using 5 point standard curves (reference standards at 0.2 to 10 times the stable isotope internal standard). Data were analyzed by 1-way ANOVA, followed by Dunnett's t posthoc analysis to compare treatments to control.

## Results

### Plasma Time Course (PPI-1011)

**Figure 1:**
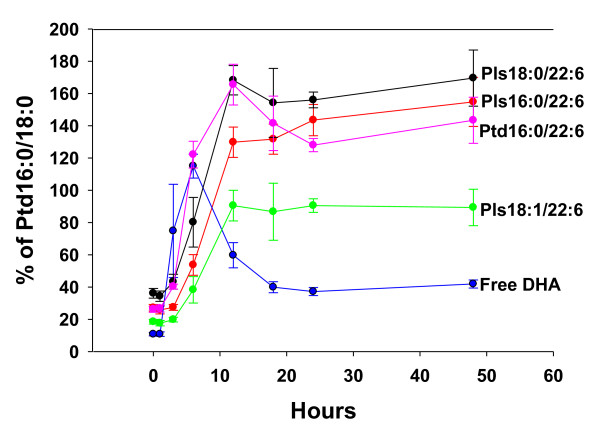
**Time course (1 to 48 hr) of conversion of PPI-1011 to free DHA and DHA-containing plasmalogens (Pls) and phosphatidylethanolamines (Ptd)**. Plasma levels are normalized to levels of the housekeeping metabolite PtdEtn 16:0/18:0. The dose of PPI-1011 was 200 mg/kg, po. N = 3 to 5 except at 12 hours (N = 7). 16:0 (palmitic acid); 18:0 (stearic acid); 18:1 (oleic acid); 22:6 (DHA).

First, PPI-1011 was not detected in plasma utilizing the MRM 815.5 ⇒ 487.3 (i.e. loss of DHA) indicating that lipase cleavage of lipoic acid at sn-3 occurs as PPI-1011 is absorbed at the gut wall. With an oral dose of 200 mg/kg, a time-dependent increase in the incorporation of PPI-1011 into circulating plasmalogens was observed (Figure 1). De-acylation at sn-2 released DHA, with maximal plasma DHA levels at 6 hours. The greatest incorporation of PPI-1011 into phospholipids was into the 16:0/22:6, 18:0/22:6 and 18:1/22:6 ethanolamine plasmalogens and phosphatidylethanolamines with maximum incorporation at 12 hours and maintenance of these levels over the remaining observation period (48 hr; Figure 1).

PPI-1011 also did not alter glycerophosphoethanolamine (GPE) plasma levels using the MRM: 214 ⇒ 140 (sn-3 phosphoethanolamine) or 16:0 lysophosphospho-ethanolamine using the MRM: 436.3 ⇒ 239.2 (sn-1 ether palmitate). These data support the conclusions that during remodeling of phospholipids derived from PPI-1011, sn-2 is rapidly reacylated following deacylation and that removal of sn-1 plus sn-2 substituents generating free glycerophosphoethanolamine is limited.

### Plasma Time Course (PPI-1038)

First, PPI-1038 was not detected in plasma utilizing the MRM 837.5 ⇒ 506.4 (i.e. loss of [^13^C_3_]DHA) indicating that lipase cleavage of lipoic acid at sn-3 occurs as PPI-1038 is absorbed at the GI wall. These samples were also spiked with PPI-1011 and the MRM 815.5 ⇒ 487.3 (i.e. loss of DHA) was clearly monitored in all samples.

**Figure 2:**
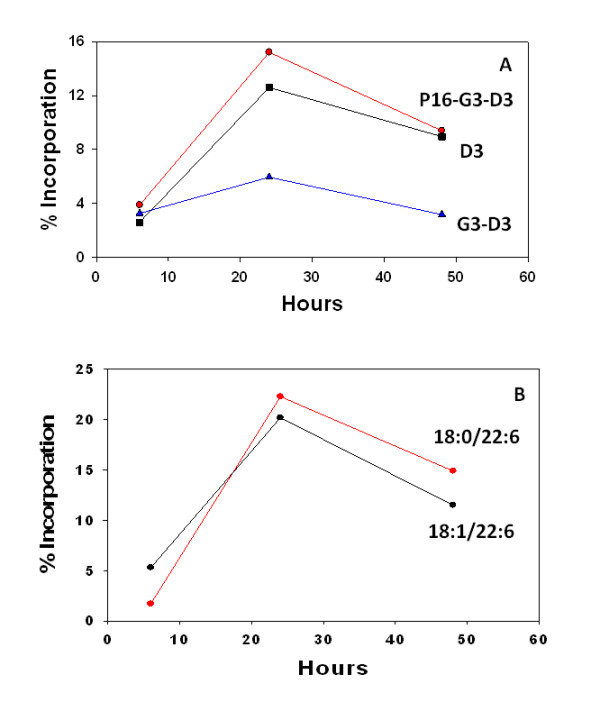
**Incorporation of [^13^C_22_]PPI-1038 into plasma DHA-containing ethanolamine plasmalogens (PlsEtn) 6, 24 and 48 hours after an oral dose of 100 mg/kg in a gelatin capsule**. N = 1 rabbit. Figure A, Labeling of PlsEtn 16:0/22:6 (D3, [^13^C_3_]DHA; G3, [^13^C_3_]glycerol; P16, [^13^C_16_]palmitic acid); Figure B, [^13^C_3_]DHA labeling of PlsEtn 18:0/22:6 and PlsEtn 18:1/22:6.

With an oral dose of 100 mg/kg, de-acylation of PPI-1038 at sn-2 released [^13^C_3_]DHA, with plasma [^13^C_3_]DHA levels constituting 40% of the plasma free DHA pool at 6 hours (Figure 2). The incorporation of [^13^C_3_]DHA into PtdEtn (16:0/22:6, 18:0/22:6 and 18:1/22:6) peaked at 24 hr. These data indicate that the increases in DHA-containing phosphatidylethanolamines observed with PPI-1011 (Figure 1) dosing are derived from increased [^13^C_3_]DHA availability and its incorporation into the sn-2 position of phosphatidylethanolamines.

**Figure 3:**
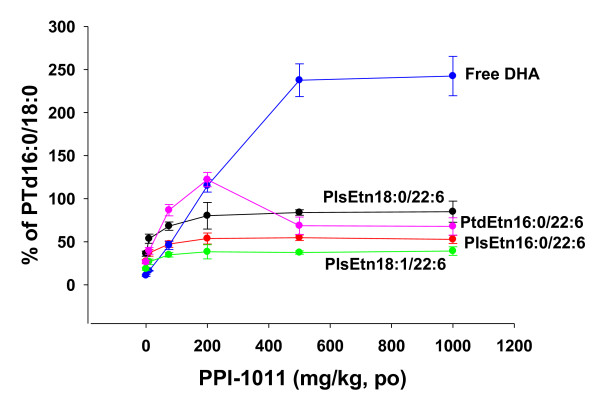
**Dose-response (10 to 1000 mg/kg) for conversion of PPI-1011 to free DHA and DHA-containing plasmalogens (PlsEtn) and phosphatidylethanolamines (PtdEtn), 6 hours post oral dosing**. Plasma levels are normalized to levels of the housekeeping metabolite PtsEtn 16:0/18:0. N = 6. 16:0 (palmitic acid); 18:0 (stearic acid); 18:1 (oleic acid); 22:6 (DHA).

Incorporation of PPI-1038 into circulating PlsEtn 16:0/22:6 was equivalent for the fully labeled form ([^13^C_16_]palmitic acid, [^13^C_3_]DHA, and [^13^C_3_]glycerol) and the [^13^C_3_]DHA-labeled form (Figure 3). In contrast, PlsEtn 18:0/22:6 and PlsEtn 18:1/22:6 were labeled with [^13^C_3_]DHA. These data support significant incorporation of released [^13^C_3_]DHA into PlsEtn 18:0/22:6 and PlsEtn 18:1/22:6 after PPI-1038 dosing.

Analysis of tissue plasmalogens 48 hours after oral dosing with PPI-1038 (100 mg/kg), demonstrated incorporation of the intact plasmalogen precursor into the target plasmalogen only in the liver (Table 1). Significant lipid remodeling occurred with both glycerol/DHA-labeled plasmalogens and DHA-labeled plasmalogens monitored in the kidney and liver. DHA-labeled plasmalogens were the major labeled plasmalogens detected in the brain (Table 1).

**Table 1:** Incorporation of stable isotopic label into tissue lipid pools in a rabbit 48 hr after an oral dose of PPI-1038.

		Plasmalogen			Phosphatidylethanolamine	
**Lipid**	**Tissue**	**P16-G3-D3 (%)**	**G3-D3 (%)**	**D3 (%)**	**G3-D3 (%)**	**D3 (%)**

16:0/22:6	Liver	15.9	5.4	10.5	0	13.2

	Kidney	0.1	1.6	18.9	0	14

	Brain	0.02	0.15	0.1	0	1

						

18:0/22:6	Liver	-	-	17.9	.84	19.8

	Kidney	-	-	22.6	.21	9.6

	Brain	-	-	0.83	0	.57

						

18:1/22:6	Liver	-	-	10	20.8	32.9

	Kidney	-	-	22.3	6.5	20.8

	Brain	-	-	3.9	2.9	1.3

						

Free DHA	Liver	-	-	24.7	-	-

	Kidney	-	-	13.6	-	-

	Brain	-	-	3.1	-	-

P16, [^13^C_16_]palmitate; G3, [^13^C_3_]glycerol; D3, [^13^C_3_]DHA.

### Plasma Dose-Response Study (PPI-1011)

Studies of dose-dependent incorporation of PPI-1011 into plasmalogens and phosphatidylethanolamines demonstrated that a new steady-state level in these circulating phospholipids was attained in a dose-dependent manner from 10 to 200 mg/kg (Figure 3). However, further increases in the dose of PPI-1011 up to 1000 mg/kg, did not further increase the steady-state levels of plasmalogens or phosphatidylethanolamines above that obtained with the 200 mg/kg dose. In contrast, the highest steady-state levels of circulating DHA were obtained at 500 mg/kg PPI-1011 and did not further increase at a dose of 1000 mg/kg (Figure 3).

### Lipoic Acid Kinetics

**Figure 4:**
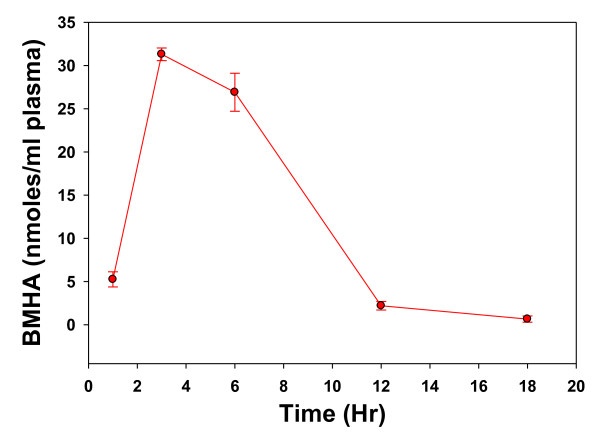
**Rabbit plasma levels of bismethylthiohexanoic acid (BMHA), a major metabolite of lipoic acid, after 200 mg/kg of PPI-1011 in gelatin capsules**. N = 4 - 6 rabbits.

Lipoic acid has a short half-life and its major metabolite, bismethythiohexanoic acid (BMHA) is a more reliable index of lipoic acid bioavailability. Rabbit plasma BMHA levels peaked 3 hr after an oral dose of 200 mg/kg, PPI-1011 (Figure 4) and declined thereafter. These data support release of lipoic acid from PPI-1011 prior to sn-2 release of DHA.

### Two week Repeated Dosing With PPI-1011

**Figure 5:**
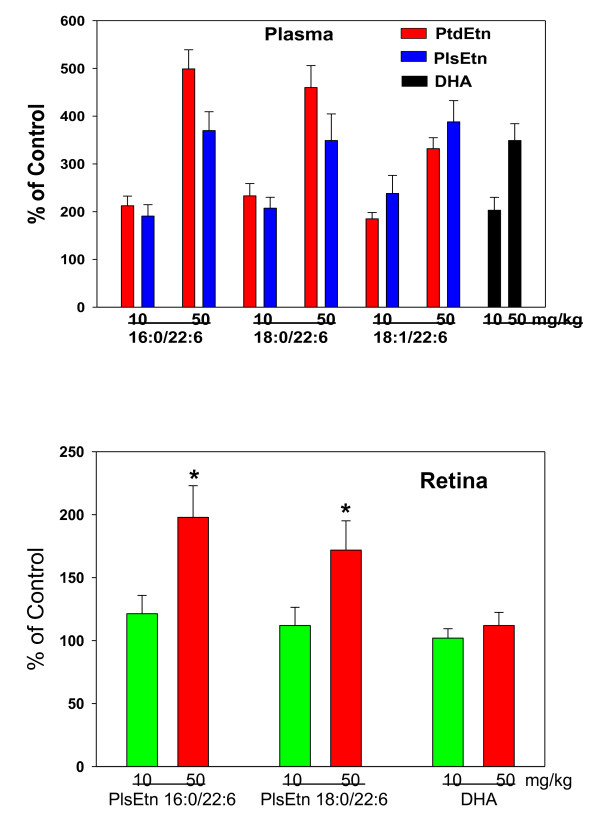
**Incorporation of PPI-1011 in retinal DHA and DHA-containing ethanolamine plasmalogens (PlsEtn) and phosphatidylethanolamines (PtdEtn) 24 hours after the last dose of a 14 day treatment with once daily dosing of 10 or 50 mg/kg in gelatin capsules**. Groups consisted of 6 rabbits.

Repeated daily oral dosing for 2 weeks with 10 or 50 mg/kg of PPI-1011 augmented circulating and retinal ethanolamine plasmalogens in a dose-dependent manner (Figure 5). This included the target plasmalogen 16:0/22:6 as well as the 18:0/22:6 and 18:1/22:6 plasmalogens. Free circulating DHA was also augmented (Figure 5).

## Discussion

Decrements in circulating DHA and plasmalogens occur early in the AD disease process [26-28] and appear to result from peroxisomal dysfunction in AD liver [24] and brain [25]. Decrements in liver and circulating DHA and plasmalogens correlate with decrements in these lipids in the brain and with cognitive decline [19-22,24,25]. To correct this plasmalogen deficit we designed a DHA-containing ether lipid precursor of plasmalogens that bypasses the requirement for peroxisomes. However, plasmalogens and ether lipids in general are unstable. In the body, plasmalogens rapidly associate with chaperone proteins after their synthesis and are not free in solution until ready for transfer to membranes. This chemical instability in the ether lipid PPI-1011 was resolved by the addition of lipoic acid at sn-3. This substituent protected the molecule from migration of the sn-2 DHA [32] but was easily cleaved by gut lipases to generate the alkyl-acyl-glycerol precursor required for the final steps of plasmalogen synthesis at the level of the endoplasmic reticulum.

PPI-1011 clearly augmented the circulating levels of the target plasmalogen (PlsEtn 16:0/22:6) in a dose-dependent and time-dependent manner. Extensive lipid remodeling occurred at sn-2 where deacylation is achieved with the 2-acyl hydrolases, phospholipase A2 (EC 3.1.1.4) and acylglycerol lipase (EC 3.1.1.23) and reacylation via a number of acyl-CoA:lysophospholipid acyltransferases [33,34] which are tightly coupled to deacylation. Deacylation coupled to reacylation of both plasmalogens and phosphatidylethanolamines by released DHA was clearly demonstrated with the stable isotope labeling study. The efficiency of this process remains to be evaluated in AD since plasmalogen-selective phospholipase A2 has been reported to be increased in AD brain [35].

With respect to supply of plasmalogen precursors to the brain, redistribution of plasmalogens synthesized by the gut epithelia [36] and the liver [23,24] is a complex process in which these lipids are exported from cells via chaperone proteins like LDL which is a major carrier of plasmalogens [37]. Tissue uptake of plasmalogens is via a LDL receptor-mediated transcytosis pathway [38,39]. In the case of the blood-brain barrier this transport pathway preferentially transports LDL enriched in DHA-containing phospholipids [40]. However, depleted levels of brain DHA, and presumably DHA-containing plasmalogens, are slowly replaced via supplementation [41]. Our labeled precursor studies support these prior observations and suggest that sustained ether lipid precursor dosing will be required to replete brain plasmalogens.

In summary, our data demonstrate that PPI-1011 is an orally bioavailable plasmalogen precursor and suggest that it may offer a new therapeutic approach for treating AD and other clinical conditions with peroxisomal dysfunction.

## List of abbreviations

16:0: palmitic acid; 18:0: stearic acid; 18:1: oleic acid; 18:2: linoleic acid; 20:4: arachidonic acid; 22:6: docosahexaeoic acid (DHA); PtdEtn: phosphatidylethanolamines; PlsEtn: plasmalogen.

## Competing interests

Authors are employees of Phenomenome Discoveries or consultants to the company.

## Authors' contributions

PLW, TS, NL, MAK, GE, and DBG all participated in the study design, supervision of assay QA/QC and data interpretation. PW, TS, NL, and GE performed experiments. PLW wrote the manuscript that was reviewed and approved by all authors.
